# Enzymatic engineering of the porcine genome with transposons and recombinases

**DOI:** 10.1186/1472-6750-7-42

**Published:** 2007-07-17

**Authors:** Karl J Clark, Daniel F Carlson, Linda K Foster, Byung-Whi Kong, Douglas N Foster, Scott C Fahrenkrug

**Affiliations:** 1 Department of Animal Science, University of Minnesota, St. Paul, MN, USA; 2 The Arnold and Mabel Beckman Center for Transposon Research, University of Minnesota, Minneapolis, MN, USA; 3 The University of Minnesota Animal Biotechnology Center, University of Minnesota, St. Paul, MN, USA

## Abstract

**Background:**

Swine is an important agricultural commodity and biomedical model. Manipulation of the pig genome provides opportunity to improve production efficiency, enhance disease resistance, and add value to swine products. Genetic engineering can also expand the utility of pigs for modeling human disease, developing clinical treatment methodologies, or donating tissues for xenotransplantation. Realizing the full potential of pig genetic engineering requires translation of the complete repertoire of genetic tools currently employed in smaller model organisms to practical use in pigs.

**Results:**

Application of transposon and recombinase technologies for manipulation of the swine genome requires characterization of their activity in pig cells. We tested four transposon systems- *Sleeping Beauty*, *Tol2*, *piggyBac*, and *Passport *in cultured porcine cells. Transposons increased the efficiency of DNA integration up to 28-fold above background and provided for precise delivery of 1 to 15 transgenes per cell. Both Cre and Flp recombinase were functional in pig cells as measured by their ability to remove a positive-negative selection cassette from 16 independent clones and over 20 independent genomic locations. We also demonstrated a Cre-dependent genetic switch capable of eliminating an intervening positive-negative selection cassette and activating GFP expression from episomal and genome-resident transposons.

**Conclusion:**

We have demonstrated for the first time that transposons and recombinases are capable of mobilizing DNA into and out of the porcine genome in a precise and efficient manner. This study provides the basis for developing transposon and recombinase based tools for genetic engineering of the swine genome.

## Background

Recent developments in livestock transgenesis, including somatic cell nuclear transfer (SCNT, cloning) [1], and stem cell biology [2,3] have energized plans to engineer the pig genome for both agricultural and emerging biomedical markets. Although pronuclear injection (PNI) and SCNT are proven methods for gene supplementation and gene targeting, respectively, more sophisticated methods for manipulating the pig genome have been lacking. Tandem gene targeting and SCNT provides a method for the precise introduction of transgenes or alternate alleles, but the inherent inefficiency of homologous recombination and donor-cell senescence limits its efficiency. Transgenesis by random integration of naked DNA has proven much more efficient for gene supplementation, whether using PNI or SCNT. However, random integration of naked DNA is often accompanied by transgene instability [4,5], transgene concatemerization [6,7], loss of transgene expression due to methylation [8-13], and short deletions, inversions and duplications at the site of transgene integration [14-25]. In addition, the lack of precision associated with random integration of naked DNA limits transgene manipulation and control post-integration.

DNA "cut and paste" transposons have been widely used for precise and efficient delivery of DNA expression cassettes into invertebrate and plant genomes. Over the past ten years, several DNA cut and paste transposon systems have been shown to function in vertebrate cells, including *Sleeping Beauty *(SB) [26,27], *Passport *(PP) [28,29], *Tol2 *[30,31], and *piggyBac *(PB) [32-34]. In addition, transposons have been used for germline transgenesis of fish [35-37], frogs [38-40], and mice [32,41-43] and for transgenesis of mouse somatic and embryonic stem cells [44-46]. It is noteworthy that although transposons function in a wide array of cell types, their efficiency can differ from species to species or even within various cell types of one species. The function and efficacy of vertebrate transposons in pig cells had not previously been examined. Demonstration that one or more transposon systems functions efficiently in porcine cells would provide a rationale for investigating their use in PNI and SCNT. In addition, the precision of transpositional transgenesis (TnT) provides a segue to the development of conditional expression systems for application in pigs and porcine cells.

Many genes have roles in multiple tissues and/or at multiple times during growth and development. Due to a requirement for strict regulation, global ectopic transgene-expression or gene-knockout will be an implausible approach for many targets. To overcome these limitations, binary systems based on transcriptional transactivation or DNA recombination have been developed and applied in model organisms for conditional gene-expression or silencing [47]. Although the tetracycline transcriptional activator system [48] has been demonstrated to function in transgenic pigs [49,50], recombinases have not. Cre and Flp recombinases catalyze a conservative DNA recombination event between two short recombinase recognition sites (RRS), *loxP *and *FRT*, respectively [51]. This results in the deletion or inversion of the DNA between two RRS- depending on their orientation. Deletion or inversion of sequences in transgenes can be used as genetic switches to activate or silence gene expression in specific cells, at particular times, or under prescribed conditions. Applications beyond conditional gene expression include the removal/recycling of selectable markers or transgenes [52] or chromosome engineering [53]. The successful application of recombinase technologies to porcine genetics requires the demonstration of Cre and/or Flp activity in porcine cells and the efficient delivery of RRS sites and recombinase-based expression vectors to the porcine genome.

In order to assess the utility of DNA transposons and recombinases for enzymatic engineering of the porcine genome, we tested four transposon systems and two recombinases. The SB, PP, *Tol2*, and PB transposon systems were able to function in cells derived from pig tissues and significantly improved the rate of transgenesis *in vitro*. Cre and Flp recombinases were capable of removing antibiotic selection cassettes in porcine cells and conditionally activating transgenes in porcine cells, demonstrating the potential for their applications to "leave no trace" and/or conditional porcine genetic engineering.

## Results

### Sleeping Beauty activity in porcine cells

**Figure 1:**
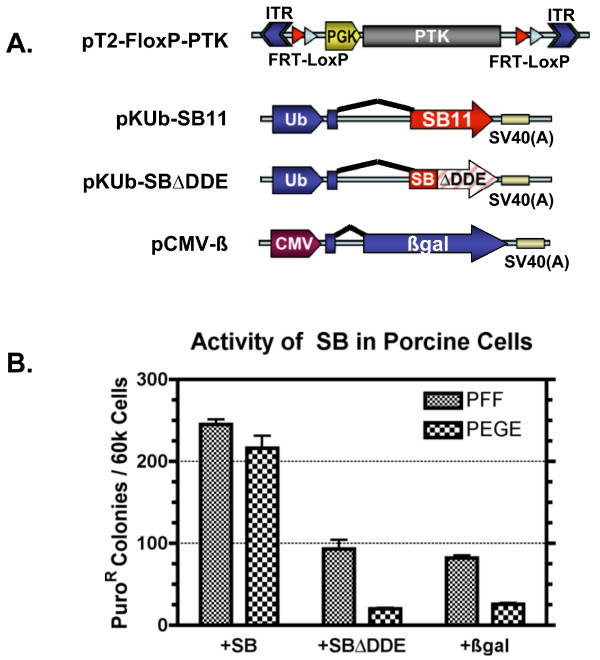
***Sleeping Beauty *function in pig cells**. A) Diagrams of the DNA vectors transfected into pig cells. pT2-FloxP-PTK is the experimental SB transposon. The transposon is flanked by inverted terminal repeats (ITR). The puromycin phosphotransferase-thymidine kinase fusion protein (PTK) is flanked by recombinase recognition sites, *FRT *and *loxP*, for Flp and Cre, respectively. pKUb-SB11 is the source of transposase and is expressed from the ubiquitin promoter (Ub). pKUb-SBΔDDE is a non-functional version of transposase because of an internal deletion within the catalytic domain. pCMV-β functions as negative control. B) The colony forming ability of pT2-FloxP-PTK in pig fetal fibroblast (PFF) and porcine endometrial gland epithelium (PEGE) was determined by counting puromycin resistant colonies after plating 60,000 cells on 10 cm dishes when pT2-FloxP-PTK was co-transfected with pKUb-SB11 (+SB), pKUb-SBΔDDE (+SBΔ*DDE*), or pCMV-β (+βgal). The addition of functional transposase (+SB) versus a non-functional transposase (SBΔDDE) or pCMV-β (Bgal) was determined to be significant by analysis with an unpaired t-test (p-values < 0.000002).

To test the ability of the SB transposon systems to mediate transposition into the porcine genome, a transposon vector (pT2-FloxP-PTK) and a transposase expression vector (pKUb-SB11) were constructed (Fig 1A). The transposon vector encodes a puromycin-thymidine kinase (PuroΔTK, PTK) fusion protein [54] between the inverted repeats of the SB transposon system. The PTK cassette was flanked by both *FRT *and *loxP *sites so that it could be used as a substrate for testing both Cre and Flp recombinases (see below). Pig fetal fibroblasts (PFF) or porcine endometrial gland epithelium (PEGE) cells were transfected with the PTK transposon along with the SB expression vector, a vector encoding non-functional SB (pKUb-SBΔDDE), or a β-galactosidase expression vector (pCMV-β). After the transfection period, cells with integrations were rendered resistant to puromycin selection, and formed clonal cell colonies after 9–12 days. Clones were stained with methylene blue and quantified (Fig. 1B). The transposase catalyzed 2.5× (PFF) -10× (PEGE) more colony formation versus transfection with a non-functional transposase (ΔDDE) or β-galactosidase. This difference in the rate of clone formation corresponds to TnT versus the background rate of non-transpositional transgenesis.

### Multiple transposon systems function in porcine cells

**Figure 2:**
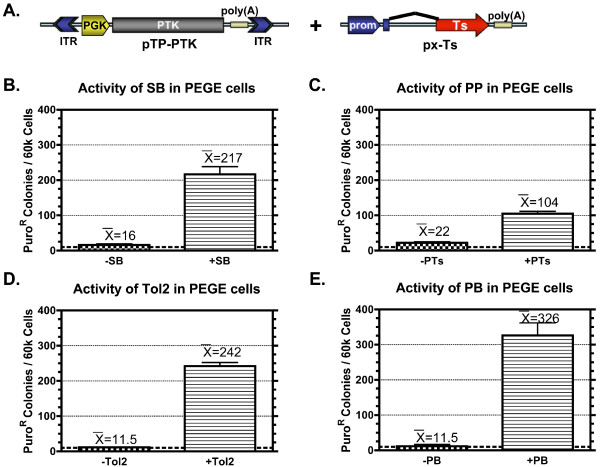
**Activity of multiple transposon systems in PEGE cells**. A) A drawing of a generic transposon (pTP-PTK) used for colony formation assays. The transposons used, except the transposon-specific inverted terminal repeats, are identical. The vector backbones of the transposons are also identical except for pGTol2P-PTK. The pKx-Ts drawing is a generic representation of the transposase-expressing vector. The promoter choices include Ub, CMV, and mCAGs for SB, *Tol2*, and PB and PP, respectively. The vector backbones and poly(A) signals are identical except for pCMV-Tol2 B-E) The number of colonies formed with SB, PP, *Tol2*, or PB PTK transposons are shown with βgal instead of transposase (-Ts) and with transposase (+Ts), where Ts is SB, PP, *Tol2*, or PB. In each case, the significance of transposase was verified with an unpaired t-test (p-values ≤ 0.00002).

The success of the SB transposon system prompted investigation of three additional transposon systems. In addition to retesting the SB transposon system in PEGE cells, we also tested PP (an additional member of the Tc1 transposon family [55])*, Tol2 *(a member of the hAT transposon family [56]), and PB, the founding member of the *piggyBac *transposon family [57]). PEGE cells are one of a few immortalized pig cell lines available, transfect consistently (8–15%), and form tight non-migrating clonal colonies- essential characteristics for the colony forming assays performed. The PTK expression cassette was placed between inverted repeats corresponding to each transposon; pKT2P-PTK, pPTnP-PTK, pGTol2P-PTK, and pPBT-PTK, respectively (Fig. 2A). PEGE cells were co-transfected with each of these transposons along with their corresponding transposase expression construct; pKUb-SB11, pKC-PTs1, pCMV-Tol2, or pKC-PB, respectively. Each transposon vector was also co-transfected with pCMV-β to determine the background rate of non-transpositional integration. Transfected PEGE cells were placed under puromycin selection for 9–12 days, colonies fixed, stained, and enumerated. Again, transfection of PEGE cells with both components of the SB system (Fig. 2B) resulted in over 200 colonies per 60,000 plated cells, or about 3.3% of transfected cells based on an average 10% transfection efficiency. This represented a 13.5-fold increase over transfection without transposase. Similar enhancements to transgenesis were seen for all the transposon systems. PP produced an average of over 100 colonies per 60,000 cells; a 5-fold increase over transfection without transposase (Fig 2C). The inclusion of *Tol2 *transposase resulted in the generation of puromycin resistant colonies at a rate 21-fold over transfections without transposase (Fig. 2D), producing on average over 240 colonies per 60,000 cells. The PB transposon system (Fig. 2E) yielded an average of over 320 colonies per 60,000 cells (about 5% of transfected cells), representing a 28-fold increase over transfection without transposase.

### Molecular characterization of transposition

**Figure 3:**
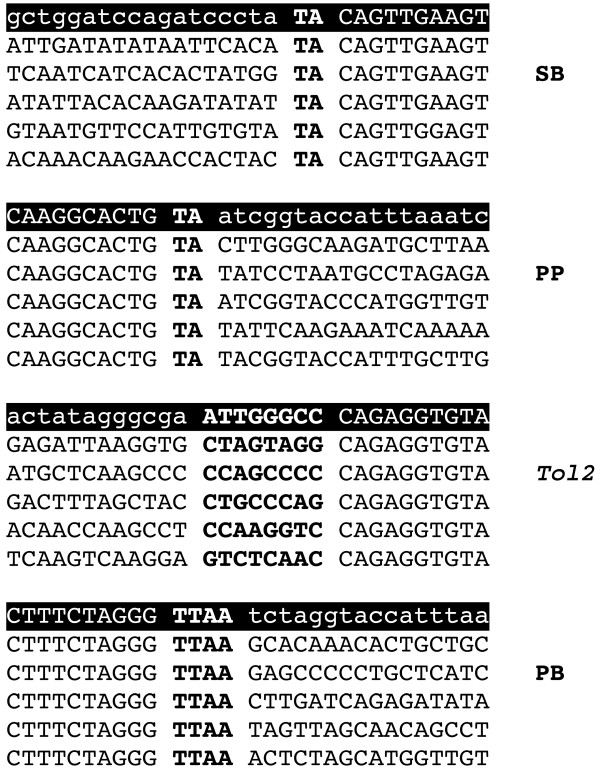
**Examples of transposon insertion junctions**. Transposon junctions amplified from PEGE cells are shown in groups of five with expected non-transposed vector sequence (lowercase) highlighted above. From top to bottom, SB (ITR-L), PP (ITR-R), *Tol2 *(ITR-L), and PB (ITR-R), and. Target site duplications (bold) for each transposon are separated from genomic DNA and corresponding (ITR) by a space.

Integration of DNA transposons produces target-site duplications upon integration into the genome. Analogous to SB and other Tc1 type transposons, the target site preference for PP is a TA dinucleotide. Target-site preference for the PB transposon is a TTAA tetranucleotide [33]. Integration of *Tol2 *results in a target-site duplication of eight bases but does not rely on specific primary sequence, instead targeting a characteristic local deformation of DNA [58]. Blocked linker-mediated PCR was used to clone junction fragments after transfection of PEGE cells with each transposon system. Characteristic integration footprints were observed for each transposon system (Fig 3). Junction sequences were compared to sequences in GenBank using BLAST [59]. Despite the small amount of contemporaneous porcine genome sequence available, some flanking DNAs of each transposon system were found to have high identity to the pig genome, in most cases in abundant repetitive elements. This demonstrates *bona fide *transposition into the porcine genome for each transposon class.

**Figure 4:**
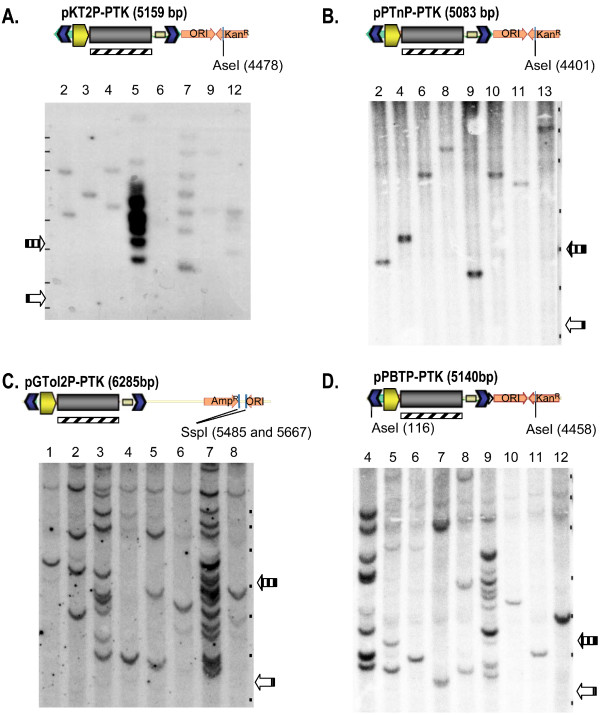
**Southern Blot of PEGE Clones**. Individual puromycin resistant PEGE colonies were isolated and expanded for Southern analysis A) SB B) PP C) *Tol2*, and D) PB. Each transposon donor plasmid transfected into PEGE cells is diagramed with restriction endonuclease sites used for DNA digestion and the probe fragment indicated (diagonal lined rectangle). Expected concatemer sizes (vertical lined arrow)/smallest possible transposition event (open arrow) for each transposon are 5159/3335 bp, 5083/3275 bp, 6285/3346 bp, and 5140/3320 bp, respectively. The positions of the marker bands are indicated by black dots on the right of each blot with sizes of 12, 10, 8, 6, 5, 4, and 3 kb are shown.

One characteristic advantage of transposase-mediated integration is the precise incorporation of one or more independently transposed gene expression cassettes, without adjacent plasmid vector. In order to observe representative integration events, DNA was isolated from 8 or 9 selected clones from each transposon and analyzed by Southern hybridization (Fig 4). Non-transposase mediated integrations, often head to tail concatemer repeats, have a predictable hybridizing fragment size following restriction enzyme digestion. However, transposon mediated events have unique DNA outside of the ITRs and therefore have unpredictable and varying fragment lengths. The enhancement of transgenesis by transposition (as detected by increased colony formation) was substantiated by the presence of inserts of varying size in cellular clones, in most cases without concatemers. The level of TnT can also be measured by counting the number of independent integrations per cellular clone. The more active transposons *Tol2 *and PB, display multiple (up to 15) independent integration events. The wild-type PP transposon system mediated a single integration event per cellular clone, reflecting its lower activity in PEGE cells, whereas the engineered SB system displayed an intermediate number of insertions.

### CRE/FLP activity in porcine cells

**Figure 5:**
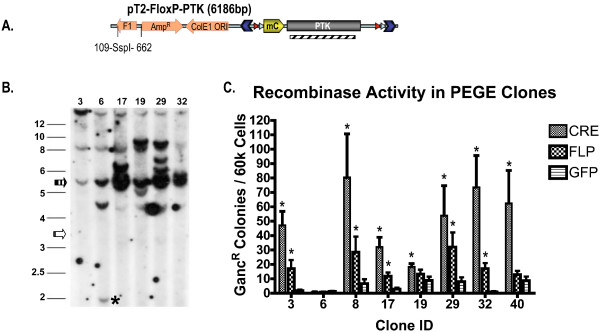
**Cre/Flp Activity in Pig Cells**. Individual puromycin resistant PEGE colonies were isolated and expanded for analysis. A) A diagram of the pT2-FloxP-PTK vector showing the location of restriction enzyme sites for SspI and the location of the PTK probe (diagonal lined rectangle). B) Southern analysis shows the number and size of vector inserts in several PEGE clones. The expected concatemer size of 5.6 kb (vertical lined arrow) as well as the smallest possible transposition event (open arrow) of 3.3 kb are indicated on the left of the image. An asterisk is placed to the right of a band slightly smaller than 2 kb in lane 2 (Clone #6). C) The rate of gancyclovir resistant colony formation after transfection of PEGE clones with pPGK-nlsCre (CRE), pKT2-nlsFlp (FLP), or pKT2C-EGFP (GFP). Values that are significantly different from the background (GFP) as determined by an unpaired t-test (p = 0.05) are designated with an asterik (*).

To test the ability of Cre and/or Flp recombinase to function in porcine cells pT2-FloxP-PTK (Fig. 1A) was transfected into PEGE cells along with SB. These clones were obtained from preliminary transfections that were selected under very stringent drug conditions that favored high-copy integrations, particularly non-transposition events. DNA from puromycin resistant clones was isolated and analyzed by Southern hybridization. Isolated clones contained multiple copies of the PTK transgene due to non-transpositional integration, as indicated by concatemers and concatemer junction bands (Fig 5). PTK transgenic clones were subsequently transfected with pPGK-nlsCre, pKT2P-nlsFlp, or pKT2C-EGFP. Excision of the PTK cassette was detectable in transiently transfected cells by PCR, and the sequence of the excision product confirmed by sequencing (data not shown). Transfected cells were placed under selection with gancyclovir for 10–14 days and colonies counted (Fig. 5C). Only cells that had excised the PTK gene could withstand gancyclovir selection. As expected for concatemers, we observed a low level of transgene instability as evidenced by the appearance of gancyclovir resistant clones upon transfection with pKT2C-EGFP. A much more pronounced recombinase stimulated elimination of the PTK cassette was demonstrated by elevated resistant colony formation for 7 out of 8 of the clones transfected with either pPGK-nlsCre or pKT2P-nlsFlp. While Cre and Flp are both active in PEGE cells, in all cases Cre mediated recombination/excision matched or exceeded that observed for Flp. A single clone (#6) never showed evidence of PTK elimination. The Southern analysis (Fig. 5B), revealed a fragment of pT2-FloxP-PTK likely resulting from the integration of a shortened PTK expression cassette lacking at least one flanking RRS. This clipped PTK transgene is therefore unable to be removed by recombinase-mediated excision.

### CRE-activated gene expression

**Figure 6:**
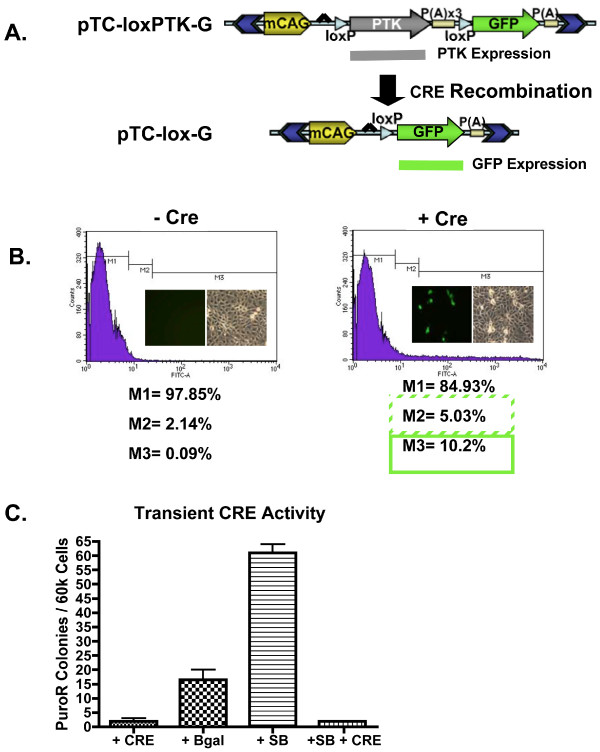
**A CRE-Activated Transgene**. A) An illustration of the Cre-activated transgene vector. The full vector, pTC-loxPTK-G, produces PTK from the mini-CAGs promoter. Transcriptional leakage into the downstream gene, GFP, is limited due to the incorporation of three full poly-adenylation signals, a so-called triple-stop. Recombination by Cre eliminates PTK and triple-stop, activating GFP expression from pTC-lox-G. B) pTC-loxPTK-G was transfected into PEGE cells with (+Cre) or without (-Cre) pPGK-nlsCre. Cells were monitored for GFP expression by fluorescent microscopy (image inserts) and flow cytometry. The percentage of cells expressing GFP was dependent on co-transfection with pPGK-nlsCre. C) PEGE cells were transfected with pTC-loxPTK-G along with pPGK-nlsCre (+Cre), pCMV-β (+βgal), pKUb-SB11 (+SB), or pKUb-SB11 and pPGK-nlsCre (+SB +Cre). The cells were plated in puromycin selective media and colonies were counted.

To further demonstrate the functionality of the transposon based Cre recombinase system for use in porcine genome engineering, a SB transposon containing a Cre-activated gene expression cassette was constructed- pTC-loxPTK-G (Fig. 6A). The PTK gene would be transcribed by the mini-CAGs promoter and efficiently terminated by three complete poly(A) signals (triple stop) in the intact pTC-loxPTK-G [60]. Cre recombination results in deletion of the PTK/triple-stop cassette, thereby juxtaposing the mini-CAGS promoter and the downstream gene expression cassette and enabling transcription of the GFP gene. Conditional activation of GFP expression was assessed by microscopy and flow cytometry after transient transfection of PEGE cells with pTC-loxPTK-G in the presence or absence of pPGK-nlsCRE (Fig. 6B). There was no GFP observed in cells transfected with pTC-loxPTK-G alone, whereas about 10–12% of the cells were GFP+ when transfected with pPGK-nlsCre. This corresponds well with the average transfection efficiency of PEGE cells, indicating that the Cre excision reaction is very efficient in transiently transfected cells.

To further examine the efficiency of Cre recombinase in transiently transfected cells, conditional removal of the PTK/triple stop expression cassette was assessed by selection in puromycin following co-transfection of PEGE cells with pTC-loxPTK-G and either Cre, β-galactosidase, SB, or Cre + SB. Transfected cells were plated under puromycin selection for 9–12 days, stained with methylene blue, and enumerated to quantify the efficiency of PTK/triple stop elimination prior to or after integration into the genome (Fig. 6C). Addition of pPGK-nlsCRE to the transfection, alone or in combination with pKUb-SB11 reduced puromycin-resistant colony counts to levels significantly lower than that observed for pKUb-SB11 or pCMV-β, which alone result in TnT and non-transpositional transgenesis with an intact PTK gene expression cassette, respectively. Therefore, Cre recombinase excision activity in transiently transfected PEGE cells approaches 100%, especially with regard to plasmids available for transposition by SB transposase.

**Figure 7:**
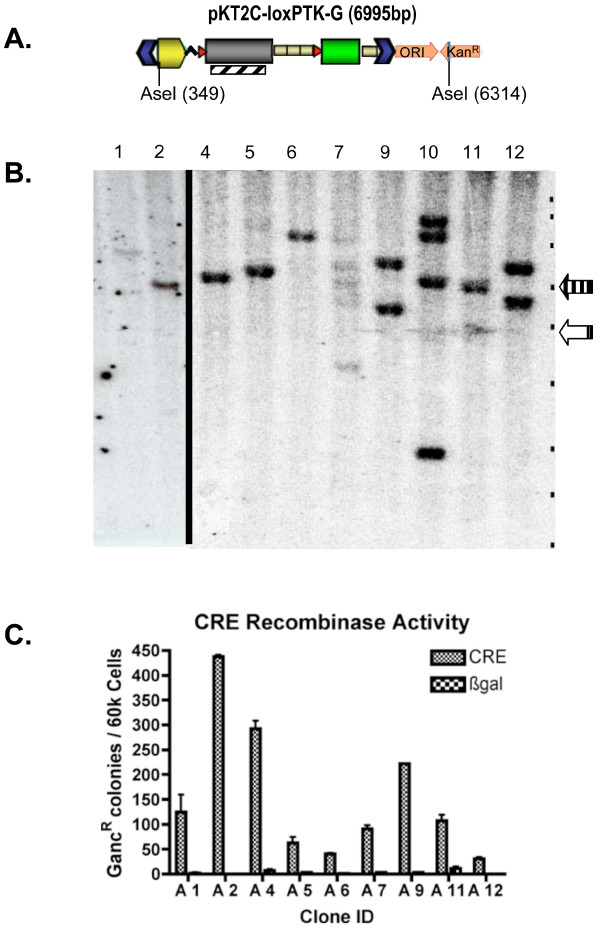
**Conditional gene-activation of integrated transposons**. Colonies from the transfection of pTC-loxPTK-G with pKUb-SB11 (Fig 5C) were expanded in selective media containing puromycin. DNA from these transgenic colonies was isolated and analyzed by Southern hybridization. A) A schematic of pKT2C-loxPTK-G that shows the AseI restriction sites and the location of the PTK hybridization probe (diagonal lined rectangle) used for Southern analysis. B) A Southern blot of pKT2C-loxPTK-G colonies. The clones were analyzed without Cre excision, so integrants that result from transposition should be equal to or greater than the transposon size of 4.9 kb (open arrow). Whereas, bands associated with concatemer formation are found at 6.0 kb (vertical line arrow). The positions of the DNA marker bands of the 1 kb Quanti-Marker from ISC Bioexpress (Kaysville, Utah), are indicated by black dots on the right of each blot with sizes of 12, 10, 8, 6, 5, 4, 3, 2.5, and 2 kb shown. C) pKT2C-loxPTK-G colonies were transfected with pPGK-nlsCre and plated under gancyclovir selection. Clones with PTK eliminated by recombination became gancyclovir resistant and were counted. Cre-activation of all clones was determined to be significant (p < 0.5).

Although this particular co-transfection with pTC-loxPTK-G and SB suffered from a low transfection efficiency (~5%) that reduced TnT (compare Fig 6C to 1B), puromycin resistant clones were expanded for characterization by Southern hybridization (Fig 7). Analysis indicated TnT with 1 to 4 transposon integrations per clone. Although, clones 7, 10 and perhaps 11 contained hybridizing species near what would be expected for non-transpositional integration, their molar representation was equal to that of single copy inserts, not multicopy concatemers. Clones 7 and 10 also harbored hybridizing species smaller than was expected for transposition. These fragments likely represent non-transposase mediated DNA recombination events. The proportion of non-transpositional integrations detected by Southern analysis (1 in 4) corresponds well with the observed unfacilitated rate of transgenesis as determined by colony count for this transfection.

pTC-loxPTK-G clones were generated to analyze the efficiency of recombinase-directed selection-cassette recycling and the conditional activation of gene expression from a variety of porcine genomic loci. Puromycin resistant clones were transfected with pPGK-nlsCRE and scored for gancylovir resistance (Fig 7C). All gancylovir resistant clones expressed GFP, although variation in the intensity of GFP was observed (data not shown) depending on the parental clone source. This expression variance is expected due to the influence of porcine sequence adjacent to the sites of transposon integration, a phenomenon commonly referred to as "position effect". A significant increase in the efficiency of selection cassette recycling was demonstrated in the presence of single copy inserts when compared to multicopy concatemers (Fig 7C vs 5C). In addition, activation of GFP expression upon recombinase-based excision from integrated transposons demonstrates the efficacy of Cre-dependent conditional gene expression in transgenic porcine cells.

## Discussion

### Multiple transposons and recombinases are active in porcine cells

This work demonstrates for the first time the capability of four DNA transposon systems, SB, *Tol2*, PB, and PP, to enzymatically facilitate precise and efficient transpositional transgenesis in porcine cells. We have also established for the first time that Cre and Flp DNA recombinases are active in porcine cells. The combination of these DNA directed enzyme systems provides for the delivery and removal of gene expression cassettes to the porcine genome for the purpose of cellular transgenesis, selection cassette recycling and conditional gene expression based on transposons and recombinases. In these studies, the *Tol2 *and PB transposon systems were more efficient than SB, which was more efficient than PP at mediating TnT in PEGE cells, although these relative efficiencies should not be over-interpreted. Although we used favorable conditions for each transposon system by our selection of promoters and transposase/transposon ratios, our focus here was on testing their function, not on determining their relative activities in PEGE cells, an immortalized cell line unsuitable for generating pigs by SCNT. Indeed, it is well established that the rate of TnT in any cell type is likely to depend not only on intrinsic transposase activity, but also on the presence or absence of cellular co-factors and DNA repair enzymes, the method of DNA introduction, and the amount of transposase produced/provided in the specific cell type. Transposon activity varies not only between cells from different species, but also between different cell types from the same species [26,27,34]. Future studies will focus on the efficiency of different transposon systems and recombinases in pig fibroblasts (applicable to SCNT), pig stem cells (for functional genomics and SCNT), and porcine embryos (for direct transgenesis by PNI).

In addition to potential differences in efficiency, the integration behavior of each transposon may be an important factor in determining the appropriate transposon system for a specific task. For instance, PB appears to preferentially integrate into transcription units [32,61]. Consistent with this observation, in our limited examination of integration sites in the pig genome, flanking sequence from two of seven PB insertions matched porcine cDNAs. In addition, PB primarily leaves no footprint when remobilized [33]. Therefore, PB may be most suitable for functional genomics studies in pigs or pig cells, where mutations due to the interruption of genes, and the potential for precise transposition-based rescue is desirable [62-65]. SB does not integrate into transcription units at a rate much higher than what would be expected by random integration [66], so it may represent a better choice for animal transgenesis, transposon-based DNA vaccination, or other somatic therapies. Alternatively transposon systems engineered to target specific genomic locations may be developed and could provide the safest choice for these applications [34,67,68]. The integration profiles of *Tol2 *and PP are not well characterized in any organism or cell type, and the integration predilections of any transposon system remains to be addressed in specific swine cells being considered for engineering.

### Advantages of transposition for pig transgenesis and genetics

There are several advantages of transpositional versus unguided transgenesis. First, the enzymatic activity of the transposase increases the efficiency of transgene integration (Fig. 1 and 2). Secondly, transposase-mediated transgenesis precisely integrates a single copy of the transposon into one or more locations in the genome. Consequently, transposition avoids the integration of G/C-rich prokaryotic elements of the vector and avoids transgene concatemerization, both of which can lead to shutdown of gene expression [5,6]. In addition, concatemerization is problematic for selection cassette recycling (Fig. 5) and the implementation of more complex genetic rearrangements with recombinases. We propose the use of transposon systems for transgenesis of porcine cells prior to their use for the creation of pigs by SCNT to enable increased efficiency, better precision, reliable expression, and selection cassette recycling. In addition, SB and PB dramatically improved the transgenesis rate in mice by PNI [32,69], providing a clear rationale for improving the efficiency of transgenic pig production via this method.

### Recombinases in swine genetics- selection-cassette recycling and conditional alleles

There are several immediate applications for recombinases in swine genetics. First, as shown in Fig 5, 6, 7, recombinases can be combined with a positive/negative selectable marker like PTK for selection cassette recycling [52]. Currently, most, if not all transgenic animals produced by SCNT contain a selectable marker (e.g. neo^R^, puro^R^, GFP) in addition to an experimental transgene. This selectable marker is useful for the proper identification of nuclear donor cells, but generally is undesirable in the transgenic animal. This could be particularly important for removal of xenogenic elements after gene knockout or manipulation preceding the introgression of engineered germplasm into agricultural production herds. The flanking of selectable markers with RRS provides the opportunity to eliminate them in culture or by breeding to Cre expressing pigs, leaving behind only a single 34-basepair RRS footprint.

Recombinases also permit the creation of conditional alleles for activation or inhibition of gene function in response to Cre or Flp recombinase activity, as illustrated in Figures 6, 7[51]. In addition, the effectiveness of homologous recombination constructs can be improved to allow selection cassette recycling, thereby avoiding 'selection cassette interference', whereby the exogenous regulatory elements in the selection cassette can interfere with the expression of genes in the vicinity of the targeted mutation [52]. As has been elegantly demonstrated in mice, recombinases can also be used to create conditional knock-outs in pigs when tissue specific ablation is desired, or when traditional knockout results in embryonic lethality. The availability of an assortment of transposon and recombinase systems should also permit serial cellular transgenesis and recombination to achieve complex genomic rearrangements in the pig. Serial transgenesis provides a direct method for the production of pigs that express several gene products. Given the dramatic long-range conservation of synteny between pig and human genomes, far more extensive than for mouse and human, engineered chromosomal rearrangements between serially provided RRS in the pig could provide superior large animal models of human congenital and cancer related chromosomal abnormalities [53].

## Conclusion

Pork represents the single most economically important meat product in the world and pigs are playing an increasingly critical role in biomedicine. An armamentarium of effective genetic tools will be required to capture the value and potential of this species for human nutrition and health. Here we have tested four transposon and two recombinase systems for activity in pig cells. SB, PP, *Tol2*, and PB and transposons are capable of precise transpositional transgenesis of porcine cells, increasing efficiency by 4–28 fold. We have also demonstrated that Cre and Flp recombinases function efficiently in the nucleus of pig cells for selection-cassette recycling and conditional regulation of transgene expression. The combination of these tools will significantly improve the efficiency and sophistication of porcine genetic manipulation for enhancing pig production and human nutrition, as well as modeling and treating human disease.

## Methods

### Vector construction

Sequence information, maps, and material requests for these constructs can be found on our web site [70].

*pT2-Floxp-PTK*- To generate a multiple cloning sequence flanked by *FRT *and *loxP *recombinase recognition sequences (*FRT*-*loxP *MCS), two oligonucleotides with overlapping sequence (shown in bold) were designed, *FRT*-*loxP *Upper [ATACCGGCCGGAAGTTCCTATTCCGAAGTTCCTATTCTCTAGAAAGTATAGGAACTTCATAACTTCGTATAATGTATGCTATACGAAGTTATCTCGAGAA**TTCCCGGGAGGCCTACTAGT**], and *FRT-loxP *Lower [GTATTCATGAGAAGTTCCTATACTTTCTAGAGAATAGGAACTTCGGAATAGGAACTTCATAACTTCGTATAGCATACATTATACGAAGTTATCCATGG**ACTAGTAGGCCTCCCGGGAA**]. These oligonucleotides were annealed and elongated by PCR using Pwo polymerase. The 218 base pair PCR fragment was cloned into pCR4 using the ZERO Blunt TOPO PCR Cloning Kit (Invitrogen, USA) to create pCR4 *FRT*-*loxP *MCS, and its sequence was verified. *FRT*-*loxP *MCS was subsequently excised with EagI and BspHI and cloned into pT2/BH [71] cleaved with EagI and NcoI to produce pT2-*FRT*-*loxP *MCS. Finally, a completely filled XhoI fragment, containing the mouse PGK promoter, the PTK fusion protein, and bovine growth hormone poly(A) signal from YTC37, a kind gift from the laboratory of A. Bradley [54], was cloned into Sma1 cleaved pT2-*FRT*-*loxP *MCS to produce pT2-FloxP-PTK.

*pKUb-SB11*- A 1.0 kb fragment of the SB11 transposase from pCMV-SB11 [72], which had been amplified with CDS-SB11-F1 [CACCATGGGAAAATCAAAAGAAATCAGCC] and CDS-SB11-R1 [GGATCCCAATTTAAAGGCAATGCTACCAAATACTAG] primers and subcloned into an intermediate vector adding a 5' BglII site and the sequence [AGATCTGAT], was cloned into the BamHI site of pKUb to make pKUb-SB11. pKUb was made by cloning nucleotides 3561–4771 of the human UbC gene (genbank accession D63791), which contains the UbC promoter, non-coding exon 1, and intron 1, into pK-SV40(A) between intact BglII and NheI restriction endonuclease sites. pK-SV40(A) was made by cloning a single copy of the SV40 poly(A) signal amplified by PCR with oligos KJC-SV40(A)-F1 [CATTGATGAGTTTGGACAAACCACA] and KJC-SV40(A)-R1 [ACCACATTTGTAGAGGTTTTACTTGCT] into pK-A10 opened with XmnI. pK-A10 was made by cloning KJC-Adapter 10 [CTGAGATCTTAAGCTAGCAGGATCCAGAATTCATTCAG] into pK digested with PvuII creating a multiple cloning site with PvuII, BglII, AflII, NheI, BamHI, EcoRI, XmnI, and PvuII recognition sites. pK was made by joining an 0.8 kb PCR product of pBluescriptSK- (Stratagene), containing the pUC_ORI amplified with oligos KJC-pUC_ORI-F1 [CTGTTCCGCTTCCTCGCTCACTGACT] and KJC-pUC_ORI-R1 [AAAAGGATCTAGGTGAAGATCCTTTTTGAT], to a 0.9 kb PCR product of pENTR-D-TOPO (Invitrogen), which contains the kanamycin resistance gene amplified by oligos KJC-KanR-F1 [CTGCATCATGAACAATAAAACTGTCTGCT] and KJC-KanR-R1 [TGCCAGTGTTACAACCAATTAACCAAT]. The junction of ORI-F1 to KanR-R1 created a single PvuII site.

*pCMV-β *is available from Clontech (Mountainview, CA).

*pPGK(nls)CRE *was a kind gift of Dr. David Largaespada's lab at the University of Minnesota.

*pKT2P-(nls)FLP*- A Flp open reading frame containing the large T antigen nuclear localization signal (bold) and a Kozak consensuses sequence was generated by amplifying the Flp open reading frame using primers CDS Kozak-NLS Flp 5' [ATATCTCGAGGCCACCATGGCT**CCCAAGAAGAAGAGGAAGGTG**ATGAGTCAATTTGATATATTATGTAAAAC] and CDS Flp 3' [ATATAGATCTTTATATGCGTCTATTTATGTAGG] using pOG44 (Invitrogen, USA) as template. The resulting PCR product was cloned into pCR4 using the ZERO Blunt TOPO PCR Cloning Kit (Invitrogen, USA) creating pCR4-nlsFlp. The nlsFlp open reading frame was subsequently excised with XhoI and BglII and inserted into XhoI-BglII cleaved pKT2-PGKi to produce pKT2P-nlsFlp. pKT2-PGKi contains the human PGK promoter, a kind gift of Dr. Scott McIvor (University of Minnesota) in front of the mini-intron, MCS, and rabbit beta-globin 3'UTR found in mini-CAGs.

*pKT2C-EGFP *was made by cloning a 0.7 kb XhoI to BglII fragment of pKT2P-GeN into pKT2-mCAG opened from BglII to XhoI. pKT2-mCAG was made by cloning a 2.2 kb BamHI to KpnI fragment of pSBT-mCAG [73] into pK-A3 opened from BamHI to KpnI. pKT2P-GeN was made by cloning EGFP as a 0.75 kb EcoRI fragment from pCR4-EGFP into the EcoRI site of pKT2P-eNeo. pCR4-EGFP was made by cloning a PCR fragment of EGFP from pEGFP-N1 (Clontech) amplified with primers KJC-EGFP-F3 [CCGAATTCTACCATGGTGAGCAAGGGCGAG] and KJC-EGFP-R2 [CCAGATCTTTACTTGTACAGCTCGTCCATGC] into pCR4-TOPO (Invitrogen). pKT2P-eNeo contains the encephalomyocarditis virus internal ribosome entry site and neomycin resistance gene amplified from pGT-Neo [62] with KJC-BactinSA-F1 [CACTGAAGTGTTGACTTCCCTGACAGC] and KJC-Bgeo-R1 [TTCAATTGTTAGAAGAACTCGTCAAGAAGGCGA]. The eNeo cassette was subcloned and acquired a modified sequence at the 3' end [GTTAACTT] to [GTTAAGTCTAGA] including a BglII site. The 1.4 kb eNeo cassette was isolated with EcoRI and BglII and moved into pKT2-PGKi opened from BglII to EcoRI.

*pKT2P-PTK *was made by cloning a 2.7 kb PvuII fragment from pKP-PTK_TS into pKT2-RV opened with EcoRV. pKT2-RV was made by cloning a 0.6 kb BamHI to KpnI fragment of pSBT-RV [73] into pK-A3 opened with BamHI and KpnI. pK-A3 was made by opening pK with PvuII and inserting KJC-Adapter 3 [CTGGATCCAGATCTGGTACCATTTAAAT] creating a small multiple cloning site with PvuII, BamHI, BglII, KpnI, and SwaI sites. pKP-PTK_TS was made by cloning a 2.3 kb BglII to EcoRI fragment of pCR4-PGK-PTK into the MCS of pK-SV40(×2) opened with EcoRI and BglII. pCR4-PGK-PTK was made by cloning a 2.3 kb PCR product of pT2-FloxP-PTK amplified with PuroΔTK-F1 [TTAGATCTGGCCTCGCACACATTCCACAT] and PuroΔTK-R1 [TGGTTCTTTCCGCCTCAGAAGCCAT] into pCR4-TOPO (Invitrogen). pK-SV40(×2) was made by cloning two copies of the SV40 poly(A) signal amplified by PCR with oligos KJC-SV40(A)-F1 [CATTGATGAGTTTGGACAAACCACA] and KJC-SV40(A)-R1 [ACCACATTTGTAGAGGTTTTACTTGCT] into pK-A10 opened with XmnI.

*pTol2-PTK*- The mini *Tol2 *transposon donor plasmid was constructed by inserting the PvuII fragment of pKP-PTK-TS into pGemT-Tol2 [74,75] opened from SwaI to HindIII (filled) to produce pGTol2P_PTK.

*pCMV-Tol2 *was constructed as indicated [74] from previously described materials [75].

*pPBTP-PTK *was made by cloning a 2.7 kb PvuII fragment of pKP-PTK_TS into pPBT-SE opened from SmaI to EcoRV. pPBT-SE was made by cloning the 102 bp PCR product containing an outward facing T7 polymerase site, the SE multiple cloning site, and an outward facing T3 polymerase site into pPBT cut with MscI. The PCR product was amplified from pKT2-SE using T7-RevComp [TCTCCCTATAGTGAGTCGTATTA] and T3-RevComp [TCTCCCTTTAGTGAGGGTTAATT] primers. pPBT was made by cloning the PB LTR1 and LTR2 into pKT2-SE from KpnI to BamHI. LTR1 and LTR 2 from PB were amplified from pXL-Bac-II, a kind gift of Malcolm Fraser (Notre Dame University), using PB-LTR1-F1 [TGGATCCCAATCCTTAACCCTAGAAAGATAATCATATTG] and PB-LTR1-R1 [GTGGCCATAAAAGTTTTGTTACTTTATAGAAG] or PB-LTR2-F1 [TTGGCCATAAGTTATCACGTAAGTAGAACATG] and PB-LTR2-R1 [TGGTACCTAGATTAACCCTAGAAAGATAGTCTG], respectively. LTR1 and LTR2 PCR products were cloned into pCR4 vector (Invitrogen) and subsequently excised by BamHI and MscI or MscI and KpnI digestion, respectively. pKT2-SE was made by cloning the 0.7 kb BamHI to KpnI fragment containing the SB inverted repeats and SE multiple cloning site from pSBT-SE [73] into pK-A3 opened from KpnI to BamHI.

*pKC-PB *was made by inserting the 2.1 kb NheI to BamHI fragment of p3XP3-DsRed, a kind gift of Dr. Malcolm Fraser (Notre Dame University), containing the PB transposase coding sequence into the 3.2 kb BamHI to NheI fragment of pKC-SB11, which resulted in the exchange of SB11 with PB transposase.

*pPTnP-PTK*- A 2.7 kb PvuII to PvuII fragment of pKP-PTK_TS was cloned into the EcoRV site of pPTn2-RV to make pPTnP-PTK. pPTn2-RV was made by cloning KJC-Adapter 4 [TCTCCCTTTAGTGAGGGTTAATTGATATCTAATACGACTCACTATAGGGAGA] into the MscI site of prePTn2(-1) creating T7 and T3 polymerase binding sites orientated out towards the inverted repeats of the PTn transposon and separated by an EcoRV site. prePPTn2(-1) was made by cloning a 0.5 kb BamHI to KpnI fragment of pCR4-PPTN2A into pK-A3 opened from KpnI to BamHI. pCR4-PPTN2A was created by topo cloning a 0.5 kb PCR product amplified from prePPTN2(-2) using oligos PPTN-F1 (BamHI) [AAGGATCCGATTACAGTGCCTTGCATAAGTAT] and PPTN-R1 (KpnI) [AAGGTACCGATTACAGTGCCTTGCATAAGTATTC] into pCR4-Topo (Invitrogen). prePPTN2(-2) was created by amplifying the majority of pBluKS-PPTN5 [29], a kind gift of Dr. Michael Leaver (University of Stirling, UK), with oligos PPTN-OL2 [CCATCTTTGTTAGGGGTTTCACAGTA] and PPTN-OR1 [CCAGGTTCTACCAAGTATTGACACA]. The PCR fragment was then self-ligated to produce an empty transposon with a single MscI site in its interior.

*pKC-PTs1 *was made by cloning a 1.0 kb NheI to EcoRI fragment of pKUb-PTs1 that contained the PPTN transposase (PTs) into pK-mCAG opened from EcoRI to NheI. pK-mCAG was made by cloning the mCAG promoter from pSBT-mCAG [73] as a 0.96 kb SmaI to EcoRI (filled) fragment into pK-SV40(A) × 2 opened with AflII (filled). pKUb-PTs1 was made by replacing the SB11 gene from pKUb-SB11 with PTs by cloning a 1.0 kb BamHI to NheI fragment from pCR4-PPTs1B into pKUb-SB11 from NheI to BamHI. pCR4-PPTs1B was made by cloning a PCR fragment of pBluKS-PPTN4 [29], a kind gift of Dr. Michael Leaver (University of Stirling, UK), amplified with primers CDS-PPTs-F1 [AAAGCTAGCATGAAGACCAAGGAGCTCACC] and CDS-PPTs-R1 [AAGGATCCTCAATACTTGGTAGAACC] into pCR4-Topo (Invitrogen).

*pKT2C-loxPTK-G *was made by cloning a 2.3 kb PvuII fragment of pK-PTK_TS into the MscI site of pKT2C-lox-GFP. pK-PTK_TS was made by cloning a 1.9 kb BglII to EcoRI fragment of pCR4-PTK into the MCS of pK-SV40(×2) opened with EcoRI and BglII. pCR4-PTK was made by cloning a 1.9 kb PCR product of pT2-FloxP-PTK using oligos PuroΔTK-F2 [TTAGATCTACCATGACCGAGTACAAGCCCA] and PuroΔTK-R1 [TGGTTCTTTCCGCCTCAGAAGCCAT] into pCR4-TOPO (Invitrogen). pKT2C-lox-GFP was made by cloning 0.1 kb EcoRI fragment of pCR4-loxP, which contains two direct repeat loxP sites separated with a MscI site, into pKT2C-EGFP opened with EcoRI. pCR4-loxP was made by topo cloning the annealed and extended oligos loxP-F1 [ATAACTTCGTATAATGTATGCTATACGAAGTTATCTCGAGTGGCCA] and loxP-R1 [ATAACTTCGTATAGCATACATTATACGAAGTTATTGGCCACTCGAG] into pCR4-TOPO (Invitrogen).

### Cell Culture and transposition/recombinase assays

Pig fibroblasts were isolated from 43 day old embryos. The tissue was dissociated using a collagenase/DNAse I treatment as well as mechanical disruption. The cells from the female piglet #8 were cultured in DMEM enriched with 10%FBS and 2× antibiotic/antimycotic solution (Gibco #15240-022). The cells were passaged in DMEM high glucose media enriched with 10% FBS, 2 mm L-glutamine, 1× P/S until spontaneously establishing line PF8. A subpopulation of porcine endometrial gland epithelium cells [76] were spontaneously immortalized, strain PEGE. The PEGE cells were maintained in DMEM supplemented with 10% FCS, 1× Penn/Strep, 10 μg/ml Insulin (Sigma, USA), and 1× L-Glutamine.

For transposition assays cells were plated in each well of a six well plate to achieve 60–80% confluence within 6–24 hours. Cells were transfected using *Trans*IT-LT1 (Mirus Bio Corporation, WI) transfection reagent according to the manufactures instructions with a ratio of 3:1 lipid: μg DNA. Each transfection contained a total of 1.15 to 1.5 μg of plasmid DNA. Wells 1–3 contained transposon plus transposase, well 4 contained transposon with no transposase, well 5 contained SB plus SB transposase and well 6 contained pKT2C-EGFP only. Molar amounts of each transposon were fixed at 1.5 × 10^-13 ^moles of transposon (0.75 × 10^-13 ^Moles for *Tol2*) while transposase plasmid was added at a molar ratio of 1:1 for SB, *Tol2*, and *PB*, and 1:0.5 for PP. The choice of the promoters and transfection ratios for SB and PP was based on the highest transposition activity observed in human HT1080 cells (data not shown). Strong promoters (CMV & miniCAGs) and transfection conditions for *Tol2 *and PB were selected based on previously published data and the observation that these transposon systems seem less susceptible to overexpression inhibition than SB and PP.[34,61,74] Total DNA weight was adjusted using pCMV-β plasmid. Forty-eight hours after transfection cells were trypsinized, and two replicates of 60,000 cells were plated onto 100 mm plates in media containing 0.3 μg/ml puromycin and selected for 9–12 days. Colonies were visualized by methylene blue staining and counted. A minimum of two six-well plates were transfected for each experiment. The mean colony number and standard error are shown in figures.

### Southern hybridizations

Several independent puromycin resistant PEGE foci for each transposon were aspirated and grown to confluence on a 100 mm plate. Genomic DNA was extracted using standard methods and approximately 10 μg was digested with SspI (*Tol2 *clones) or AseI (SB, PB, and PP) clones. Digested DNA was separated on 0.7% agarose gel and transferred to positively charged nylon membranes (GE Osmotics, USA). Membranes were probed with a random primed 1524 bp XmaI fragment of pKP-PTK-TS that contained the bulk of the PTK gene and visualized by autoradiography or phosphor imaging.

### Cloning transposon junctions

Genomic DNA was isolated from pooled, fixed, and stained puromycin resistant clones for each transposon. For splinkerette PCR DNA was cut with Sau3AI or NlaIII and junctions were cloned as described [69]. For blocked linker-mediated PCR, DNA was cut with NspI for Tol2 and SB, and a cocktail of enzymes including XbaI, AvrII, NheI and SpeI for PB and PP. The NspI digested DNA was ligated to the blocked linker-SphI that was created by annealing primerette-long [CCTCCACTACGACTCACTGAAGGGCAAGCAGTCCTAACAACCATG] and blink-SphI [5'P- GTTGTTAGGACTGCTTGC-3'P]. Whereas the DNA digested with the cocktail was ligated to the blocked linker-XbaI that was produced by annealing primerette long to blink-XbaI [5'P-CTAGCATGGTTGTTAGGACTGCTTGC-3'P]. Following ligation the junction sequences were amplified by nested PCR. The primary PCR used the common primer primerette-short [CCTCCACTACGACTCACTGAAGGGC] with transposon-specific primers SB_IRDR(L)-O1 [ATTTTCCAAGCTGTTTAAAGGCACAGTCAAC], Tol2(L)-O1 [AATTAAACTGGGCATCAGCGCAATT], PB-LTR(R)-O1 [ACAGACCGATAAAACACATGCGTCAA], and PTn-IRDR(R)-O1 [GGGTGAATACTTATGCACCCAACAGATG]. The secondary PCR reactions used the common primer primerette-nested [GGGCAAGCAGTCCTAACAACCATG] with transposon-specific primers SB_IRDR(L)-O2 [GACTTGTGTCATGCACAAAGTAGATGTCCT], Tol2(L)-O2 [GCGCAATTCAATTGGTTTGGTAATAGC], PB-LTR(R)-O2 [TCCTAAATGCACAGCGACGGATTC], and PTn-IRDR(R)-O2 [CAGTACATAATGGGAAAAAGTCCAAGGG]. To generate unique sequences serial dilutions (1:50 and 1:500) of the ligation reaction were used as template for the primary PCR. The primary PCR was diluted 1:50 and used as template in the secondary PCR reaction. The PCR fragments were shotgun cloned and sequenced.

## Abbreviations

PEGE Porcine endometrial glandular epithelium

GFP Green fluorescent protein

RRS Recombinase recognition site

SCNT Somatic cell nuclear transfer

PNI Pronuclear microinjection

ITR Inverted terminal repeat

SB *Sleeping Beauty*

PP *Passport*

PB *piggyBac*

TnT transpositional transgenesis

## Authors' contributions

KJC designed experiments and transposon vectors and together with DFC constructed transposons, performed experiments, and conducted molecular analysis. LKF optimized and conducted tissue culture experiments. BWK performed preliminary transfections in pig cells and was mentored by DNF. SCF conceived the study and mentored KJC, DFC, and LKF in experimental design and data analysis. KJC drafted the manuscript and along with DFC and SCF completed manuscript preparation. All authors read and approved the final manuscript.

## References

[B1] Lai L, Prather RS (2003). Creating genetically modified pigs by using nuclear transfer. Reprod Biol Endocrinol.

[B2] Zeng L, Rahrmann E, Hu Q, Lund T, Sandquist L, Felten M, O'Brien TD, Zhang J, Verfaillie C (2006). Multipotent adult progenitor cells from swine bone marrow. Stem cells (Dayton, Ohio).

[B3] Price EM, Prather RS, Foley CM (2006). Multipotent adult progenitor cell lines originating from the peripheral blood of green fluorescent protein transgenic Swine. Stem Cells Dev.

[B4] Pravtcheva DD, Wise TL (2003). Transgene instability in mice injected with an in vitro methylated Igf2 gene. Mutat Res.

[B5] Scrable H, Stambrook PJ (1999). A genetic program for deletion of foreign DNA from the mammalian genome. Mutat Res.

[B6] Garrick D, Fiering S, Martin DI, Whitelaw E (1998). Repeat-induced gene silencing in mammals. Nat Genet.

[B7] Henikoff S (1998). Conspiracy of silence among repeated transgenes. Bioessays.

[B8] Dalle B, Rubin JE, Alkan O, Sukonnik T, Pasceri P, Yao S, Pawliuk R, Leboulch P, Ellis J (2005). eGFP reporter genes silence LCRbeta-globin transgene expression via CpG dinucleotides. Mol Ther.

[B9] Hammer RE, Pursel VG, Rexroad CE, Wall RJ, Bolt DJ, Ebert KM, Palmiter RD, Brinster RL (1985). Production of transgenic rabbits, sheep and pigs by microinjection. Nature.

[B10] Krumlauf R, Hammer RE, Tilghman SM, Brinster RL (1985). Developmental regulation of alpha-fetoprotein genes in transgenic mice. Mol Cell Biol.

[B11] Ornitz DM, Palmiter RD, Hammer RE, Brinster RL, Swift GH, MacDonald RJ (1985). Specific expression of an elastase-human growth hormone fusion gene in pancreatic acinar cells of transgenic mice. Nature.

[B12] Palmiter RD, Norstedt G, Gelinas RE, Hammer RE, Brinster RL (1983). Metallothionein-human GH fusion genes stimulate growth of mice. Science.

[B13] Townes TM, Chen HY, Lingrel JB, Palmiter RD, Brinster RL (1985). Expression of human beta-globin genes in transgenic mice: effects of a flanking metallothionein-human growth hormone fusion gene. Mol Cell Biol.

[B14] Mark WH, Signorelli K, Blum M, Kwee L, Lacy E (1992). Genomic structure of the locus associated with an insertional mutation in line 4 transgenic mice. Genomics.

[B15] Chen CM, Choo KB, Cheng WT (1995). Frequent deletions and sequence aberrations at the transgene junctions of transgenic mice carrying the papillomavirus regulatory and the SV40 TAg gene sequences. Transgenic research.

[B16] Pravtcheva DD, Wise TL (1995). A postimplantation lethal mutation induced by transgene insertion on mouse chromosome 8. Genomics.

[B17] Nakanishi T, Kuroiwa A, Yamada S, Isotani A, Yamashita A, Tairaka A, Hayashi T, Takagi T, Ikawa M, Matsuda Y (2002). FISH analysis of 142 EGFP transgene integration sites into the mouse genome. Genomics.

[B18] Bishop JO, Houdebine L-M (1997). Chromosomal Insertion of Foreign DNA. Transgenic animals: generation and use.

[B19] Covarrubias L, Nishida Y, Mintz B (1986). Early postimplantation embryo lethality due to DNA rearrangements in a transgenic mouse strain. Proc Natl Acad Sci USA.

[B20] Gordon JW, Ruddle FH (1985). DNA-mediated genetic transformation of mouse embryos and bone marrow – a review. Gene.

[B21] Hamada T, Sasaki H, Seki R, Sakaki Y (1993). Mechanism of chromosomal integration of transgenes in microinjected mouse eggs: sequence analysis of genome-transgene and transgene-transgene junctions at two loci. Gene.

[B22] Overbeek PA, Lai SP, Van Quill KR, Westphal H (1986). Tissue-specific expression in transgenic mice of a fused gene containing RSV terminal sequences. Science.

[B23] Rohan RM, King D, Frels WI (1990). Direct sequencing of PCR-amplified junction fragments from tandemly repeated transgenes. Nucleic acids research.

[B24] Takano M, Egawa H, Ikeda JE, Wakasa K (1997). The structures of integration sites in transgenic rice. Plant J.

[B25] Wilkie TM, Palmiter RD (1987). Analysis of the integrant in MyK-103 transgenic mice in which males fail to transmit the integrant. Mol Cell Biol.

[B26] Ivics Z, Hackett PB, Plasterk RH, Izsvak Z (1997). Molecular reconstruction of Sleeping Beauty, a Tc1-like transposon from fish, and its transposition in human cells. Cell.

[B27] Izsvak Z, Ivics Z, Plasterk RH (2000). Sleeping Beauty, a wide host-range transposon vector for genetic transformation in vertebrates. J Mol Biol.

[B28] Clark KJ, Leaver MJ, Foster LK, Carlson DF, Fahrenkrug SC Passport, a native Tc1/mariner transposon from Pleuronectes platessa, functions in vertebrate cells. in preparation.

[B29] Leaver MJ (2001). A family of Tc1-like transposons from the genomes of fishes and frogs: evidence for horizontal transmission. Gene.

[B30] Koga A, Iida A, Kamiya M, Hayashi R, Hori H, Ishikawa Y, Tachibana A (2003). The medaka fish Tol2 transposable element can undergo excision in human and mouse cells. J Hum Genet.

[B31] Koga A, Suzuki M, Inagaki H, Bessho Y, Hori H (1996). Transposable element in fish. Nature.

[B32] Ding S, Wu X, Li G, Han M, Zhuang Y, Xu T (2005). Efficient transposition of the piggyBac (PB) transposon in mammalian cells and mice. Cell.

[B33] Fraser MJ, Ciszczon T, Elick T, Bauser C (1996). Precise excision of TTAA-specific lepidopteran transposons piggyBac (IFP2) and tagalong (TFP3) from the baculovirus genome in cell lines from two species of Lepidoptera. Insect Mol Biol.

[B34] Wu SC, Meir YJ, Coates CJ, Handler AM, Pelczar P, Moisyadi S, Kaminski JM (2006). piggyBac is a flexible and highly active transposon as compared to sleeping beauty, Tol2, and Mos1 in mammalian cells. Proc Natl Acad Sci USA.

[B35] Davidson AE, Balciunas D, Mohn D, Shaffer J, Hermanson S, Sivasubbu S, Cliff MP, Hackett PB, Ekker SC (2003). Efficient gene delivery and gene expression in zebrafish using the Sleeping Beauty transposon. Dev Biol.

[B36] Kawakami K, Koga A, Hori H, Shima A (1998). Excision of the tol2 transposable element of the medaka fish, Oryzias latipes, in zebrafish, Danio rerio. Gene.

[B37] Kawakami K, Shima A, Kawakami N (2000). Identification of a functional transposase of the Tol2 element, an Ac-like element from the Japanese medaka fish, and its transposition in the zebrafish germ lineage. Proc Natl Acad Sci USA.

[B38] Hamlet MR, Yergeau DA, Kuliyev E, Takeda M, Taira M, Kawakami K, Mead PE (2006). Tol2 transposon-mediated transgenesis in Xenopus tropicalis. Genesis.

[B39] Kawakami K, Imanaka K, Itoh M, Taira M (2004). Excision of the Tol2 transposable element of the medaka fish Oryzias latipes in Xenopus laevis and Xenopus tropicalis. Gene.

[B40] Sinzelle L, Vallin J, Coen L, Chesneau A, Du Pasquier D, Pollet N, Demeneix B, Mazabraud A (2006). Generation of trangenic Xenopus laevis using the Sleeping Beauty transposon system. Transgenic research.

[B41] Dupuy AJ, Fritz S, Largaespada DA (2001). Transposition and gene disruption in the male germline of the mouse. Genesis.

[B42] Fischer SE, Wienholds E, Plasterk RH (2001). Regulated transposition of a fish transposon in the mouse germ line. Proc Natl Acad Sci USA.

[B43] Horie K, Kuroiwa A, Ikawa M, Okabe M, Kondoh G, Matsuda Y, Takeda J (2001). Efficient chromosomal transposition of a Tc1/mariner- like transposon Sleeping Beauty in mice. Proc Natl Acad Sci USA.

[B44] Kawakami K, Noda T (2004). Transposition of the Tol2 element, an Ac-like element from the Japanese medaka fish Oryzias latipes, in mouse embryonic stem cells. Genetics.

[B45] Luo G, Ivics Z, Izsvak Z, Bradley A (1998). Chromosomal transposition of a Tc1/mariner-like element in mouse embryonic stem cells. Proc Natl Acad Sci USA.

[B46] Yant SR, Meuse L, Chiu W, Ivics Z, Izsvak Z, Kay MA (2000). Somatic integration and long-term transgene expression in normal and haemophilic mice using a DNA transposon system. Nat Genet.

[B47] Lewandoski M (2001). Conditional control of gene expression in the mouse. Nat Rev Genet.

[B48] Gossen M, Bujard H (1992). Tight control of gene expression in mammalian cells by tetracycline-responsive promoters. Proc Natl Acad Sci USA.

[B49] Choi BR, Koo BC, Ahn KS, Kwon MS, Kim JH, Cho SK, Kim KM, Kang JH, Shim H, Lee H (2006). Tetracycline-inducible gene expression in nuclear transfer embryos derived from porcine fetal fibroblasts transformed with retrovirus vectors. Mol Reprod Dev.

[B50] Kues WA, Schwinzer R, Wirth D, Verhoeyen E, Lemme E, Herrmann D, Barg-Kues B, Hauser H, Wonigeit K, Niemann H (2006). Epigenetic silencing and tissue independent expression of a novel tetracycline inducible system in double-transgenic pigs. Faseb J.

[B51] Branda CS, Dymecki SM (2004). Talking about a revolution: The impact of site-specific recombinases on genetic analyses in mice. Dev Cell.

[B52] Abuin A, Bradley A (1996). Recycling selectable markers in mouse embryonic stem cells. Mol Cell Biol.

[B53] Yu Y, Bradley A (2001). Engineering chromosomal rearrangements in mice. Nat Rev Genet.

[B54] Chen YT, Bradley A (2000). A new positive/negative selectable marker, puDeltatk, for use in embryonic stem cells. Genesis.

[B55] Plasterk RH, Izsvak Z, Ivics Z (1999). Resident aliens: the Tc1/mariner superfamily of transposable elements. Trends Genet.

[B56] Kempken F, Windhofer F (2001). The hAT family: a versatile transposon group common to plants, fungi, animals, and man. Chromosoma.

[B57] Sarkar A, Sim C, Hong YS, Hogan JR, Fraser MJ, Robertson HM, Collins FH (2003). Molecular evolutionary analysis of the widespread piggyBac transposon family and related "domesticated" sequences. Mol Genet Genomics.

[B58] Hackett CS, Geurts AM, Wangensteen KJ, Balciunas D, Ekker SC, Hackett PB Predicting transposon chromosomal insertion sites: implications for functional genomics and gene therapy. Genome Biology.

[B59] Altschul SF, Gish W, Miller W, Myers EW, Lipman DJ (1990). Basic local alignment search tool. J Mol Biol.

[B60] Vallier L, Mancip J, Markossian S, Lukaszewicz A, Dehay C, Metzger D, Chambon P, Samarut J, Savatier P (2001). An efficient system for conditional gene expression in embryonic stem cells and in their in vitro and in vivo differentiated derivatives. Proc Natl Acad Sci USA.

[B61] Wilson MH, Coates CJ, George AL (2007). PiggyBac Transposon-mediated Gene Transfer in Human Cells. Mol Ther.

[B62] Clark KJ, Geurts AM, Bell JB, Hackett PB (2004). Transposon vectors for gene-trap insertional mutagenesis in vertebrates. Genesis.

[B63] Collier LS, Carlson CM, Ravimohan S, Dupuy AJ, Largaespada DA (2005). Cancer gene discovery in solid tumours using transposon-based somatic mutagenesis in the mouse. Nature.

[B64] Dupuy AJ, Akagi K, Largaespada DA, Copeland NG, Jenkins NA (2005). Mammalian mutagenesis using a highly mobile somatic Sleeping Beauty transposon system. Nature.

[B65] Geurts AM, Wilber A, Carlson CM, Lobitz PD, Clark KJ, Hackett PB, McIvor RS, Largaespada DA (2006). Conditional gene expression in the mouse using a Sleeping Beauty gene-trap transposon. BMC Biotechnol.

[B66] Yant SR, Wu X, Huang Y, Garrison B, Burgess SM, Kay MA (2005). High-resolution genome-wide mapping of transposon integration in mammals. Mol Cell Biol.

[B67] Ivics Z, Katzer A, Stuwe EE, Fiedler D, Knespel S, Izsvak Z (2007). Targeted sleeping beauty transposition in human cells. Mol Ther.

[B68] Yant SR, Huang Y, Akache B, Kay MA (2007). Site-directed transposon integration in human cells. Nucleic acids research.

[B69] Dupuy AJ, Clark K, Carlson CM, Fritz S, Davidson AE, Markley KM, Finley K, Fletcher CF, Ekker SC, Hackett PB (2002). Mammalian germ-line transgenesis by transposition. Proc Natl Acad Sci USA.

[B70] Fahrenkrug Lab Home Page. http://primer.ansci.umn.edu/Fahrenkruglab.

[B71] Hackett Lab Plasmid Info. http://www.cbs.umn.edu/labs/perry/plasmids/plasmid.html.

[B72] Geurts AM, Yang Y, Clark KJ, Liu G, Cui Z, Dupuy AJ, Bell JB, Largaespada DA, Hackett PB (2003). Gene transfer into genomes of human cells by the sleeping beauty transposon system. Mol Ther.

[B73] Ohlfest JR, Frandsen JL, Fritz S, Lobitz PD, Perkinson SG, Clark KJ, Nelsestuen G, Key NS, McIvor RS, Hackett PB (2005). Phenotypic correction and long-term expression of factor VIII in hemophilic mice by immunotolerization and nonviral gene transfer using the Sleeping Beauty transposon system. Blood.

[B74] Balciunas D, Wangensteen KJ, Wilber A, Bell J, Geurts A, Sivasubbu S, Wang X, Hackett PB, Largaespada DA, McIvor RS (2006). Harnessing a high cargo-capacity transposon for genetic applications in vertebrates. PLoS Genet.

[B75] Parinov S, Kondrichin I, Korzh V, Emelyanov A (2004). Tol2 transposon-mediated enhancer trap to identify developmentally regulated zebrafish genes in vivo. Dev Dyn.

[B76] Deachapunya C, Palmer-Densmore M, O'Grady SM (1999). Insulin stimulates transepithelial sodium transport by activation of a protein phosphatase that increases Na-K ATPase activity in endometrial epithelial cells. J Gen Physiol.

